# Unraveling the functional signals of rods and cones in the human retina: separation and analysis

**DOI:** 10.3389/fopht.2024.1340692

**Published:** 2024-04-08

**Authors:** Clara Pfäffle, Léo Puyo, Hendrik Spahr, Dierck Hillmann, Yoko Miura, Gereon Hüttmann

**Affiliations:** ^1^ Institute of Biomedical Optics, University of Lübeck, Lübeck, Germany; ^2^ Medical Laser Center Lübeck GmbH, Lübeck, Germany; ^3^ Department of Physics, Faculty of Science, Vrije Universiteit Amsterdam, Amsterdam, Netherlands; ^4^ Department of Ophthalmology, University of Lübeck, Lübeck, Germany; ^5^ Airway Research Center North (ARCN), University of Lübeck, German Center for Lung Research (DZL), Lübeck, Germany

**Keywords:** optical cohererence tomography (OCT), optoretinography (ORG), phase-sensitive OCT, retinal imaging, functional imaging, rods, cones

## Abstract

In recent years, optoretinography has become an important functional imaging method for the retina, as light-evoked changes in the photoreceptors have been demonstrated for a large number of different OCT systems. Full-field swept-source optical coherence tomography (FF-SS-OCT) is particularly phase-stable, and it is currently the only technique sensitive enough to detect the smaller functional changes in the inner plexiform layer (IPL). However, the resolution of state-of-the art FF-SS-OCT systems is not high enough to distinguish individual photoreceptors. This makes it difficult to separate rods from cones. In this work, we circumvent this problem by separating the functional changes in rods and cones by their different temporal dynamics to the same light stimulus. For this purpose, a mathematical model was developed that represents the measured signals as a superposition of two impulse responses. The developed model describes the measured data under different imaging conditions very well and is able to analyze the sensitivity and temporal dynamics of the two photoreceptor types separately.

## 1 Introduction

The study of the functional characteristics of photoreceptors is of great importance in the context of a wide range of retinal diseases, covering both diagnostic and therapeutic aspects. As a result, a plethora of different imaging modalities has emerged in recent years, utilizing different contrast mechanisms such as changes in reflectance spectra (1–3) or scattering behavior (4–6), each designed to capture the nuanced intricacies of photoreceptor function. An emerging functional imaging approach that has gained prominence uses the optical phase of the backscattered light in optical coherence tomography (OCT) data (7–11), and is known as optoretinography (ORG). This method has attracted considerable attention due to its high sensitivity and inherent robustness.

Phase stability for ORG measurements can be achieved either by very fast scanning of the sample or by fully parallel imaging, as is done in full-field swept-source OCT (FF-SS-OCT). By simultaneously recording the FF-SS-OCT data, the phases of the detected signal can be analyzed in a much more robust way over time, allowing changes in the retina to be detected with greater sensitivity. As a result, FF-SS-OCT is so far the only imaging technique that can measure the small functional changes in the inner plexiform layer (IPL) in the living human retina, in addition to the functional signals in the photoreceptor cells (12, 13). This makes it possible to study signal processing in the retina.

However, scanning systems are superior to FF-SS-OCT in both lateral and axial resolution. This is achieved through the confocal gating and through the availability of light sources with a wider bandwidth, respectively. For these reasons, scanning systems are particularly well suited for single-cell measurements of individual cones and rods. In combination with adaptive optics and scanning laser ophthalmoscopy (SLO) even individual rods can be resolved (14). Thus, the functional contributions of rods and cones can be separated directly with SLO based optoretinography measurements.

**Figure 1:**
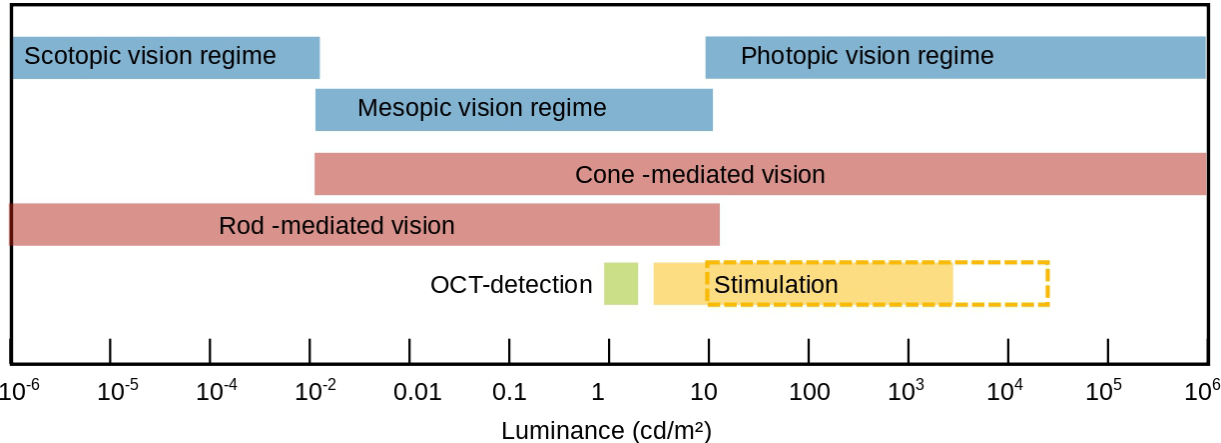
Regimes of scotopic, mesopic and photopic vision in luminance. The Luminance values for scotopic, mesoscopic, photopic rod dominated, and cone dominated vision were taken from the work of Zele and Cao (15). The green bar corresponds to the luminance caused by the OCT detection. The filled yellow bar corresponds to the calculated luminances used here for stimulation. The yellow dashed framed bar corresponds to the luminance considering the pupil diameter (see Section 4).

This is not possible with FF-SS-OCT, as both lateral and axial resolution are not yet sufficient to resolve rods or separate their tips from those of the cones. Instead, FF-SS-OCT detects a combination of functional changes in both cell types in the ORG signal. Photoreceptors are typically stimulated at intensities in the highly photopic range, where cone activity dominates (10 cd*/*m^2^ to 10^6^ cd*/*m^2^). Thus, ignoring the rod contribution to the signal only leads to a negligible error. Rods are more sensitive to low-light conditions (10^−6^ cd*/*m^2^ to 10^−2^ cd*/*m^2^ (15)), where cones are either not functioning or less sensitive due to a less powerful amplification cascade (**Figure 1**). For this reason, their contribution becomes more pronounced at lower stimulation intensities.

These low stimulation intensities for rod-dominated signals cannot be realized with high-resolution OCT systems, which have to work at a wavelength around 850 nm. In this wavelength range, the human retina is still sensitive, resulting in a stimulation of the retina by the OCT measurement itself (the local illumination by an OCT beam with wavelengths around 850 nm corresponds to luminances in the single digit cd*/*m^2^ range). ORG measurements are limited here to higher stimulation powers, making it difficult to measure rods and cones separately.

Besides their different sensitivity to different light levels, rods and cones also differ in the dynamics with which they respond to light stimulation (16, 17). In this study, we have taken advantage of this difference in the temporal behavior of rod and cone photoreceptors to separate their respective contributions to the functional signal without requiring any spatial separation of their responses. As a result, it is no longer necessary to resolve the individual cones and rods laterally or axially, greatly reducing the requirements on the imaging system.

## 2 Results

**Figure 2:**
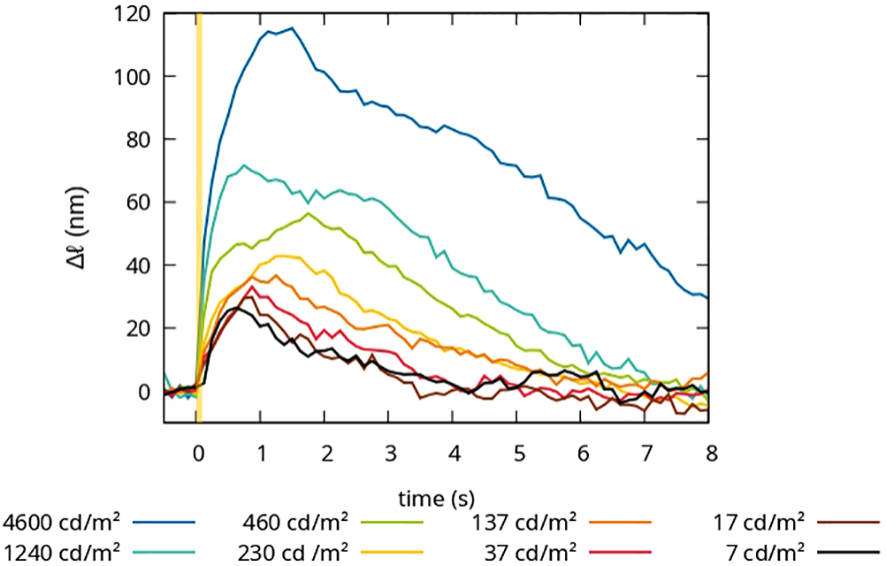
Time courses of the optical path length changes for stimuli of 100ms duration and different luminances.

The optoretinography (ORG) measurements here were performed with an FF-SS-OCT setup on a healthy volunteer. The stimulation intensities were in the mesoscopic range, where rods and cones are equally active. To better distinguish the responsiveness of both rod and cone photoreceptors to variations in intensity, different optical density (OD) filters were introduced into the stimulation pathway. This manipulation produces irradiance levels between 1.92 *µ*W*/*mm^2^ and 3 nW*/*mm^2^ at the retinal level. To facilitate comparison with existing literature and physiological phenomena, the photon flux, luminance, and degree of photoreceptor bleaching were calculated (see **Table 1**). The duration of each measurement was 8.6 seconds. The stimulation was triggered from the beginning of the fifth volume within this measurement sequence and lasted 100 milliseconds. The temporal change in optical path length for varying luminance conditions is succinctly depicted in **Figure 2**. Obviously, the rise time and shape of the transients change with the stimulation level. At 137 cd*/*m^2^ and below a single pulse-shaped transient is observed. At higher luminance, first a shoulder and then a second peak appear at the rising edge of the transient.

**Table 1:** List of the used stimulation intensities expressed in intensity, luminance, photon flux and the percentage of photopigment bleaching per second (PPB R) in rods and (PPB C) in cones.

OD-filter	Irradiance $\left[\frac{\text{µW}}{{\text{mm}}^{2}}\right]$	$\frac{\text{photons}}{\text{μ}{\text{m}}^{2}\times \text{s}}\left[\times 10^{6}\right]$	luminance $\left[\frac{\text{cd}}{{\text{m}}^{2}}\right]$	PPB R $\left[\frac{\%}{\text{s}}\right]$	PPB C $\left[\frac{\%}{\text{s}}\right]$
0.0	1.9	5.3	4600	10.0	40.0
0.6	0.52	1.44	1240	3.2	12.8
1.0	0.19	0.53	460	1.0	4.0
1.3	96.0 × 10^−3^	0.27	230	0.5	2.0
1.6	57.0 × 10^−3^	0.16	137	0.3	1.2
2.0	15.4 × 10^−3^	0.04	37	0.08	0.32
2.3	7.0 × 10^−3^	0.02	17	0.04	0.16
2.6	3.0 × 10^−3^	0.008	7	0.02	0.08

Further insight can be gained by analyzing the composite character of the measured temporal profiles by assuming a combination of two distinct curves. This distinction suggests that the observed functional response can be explained as the result of two physiological processes. One process, dominates at low stimulation levels, the other process is characterized by a rapid onset at higher stimulation intensities, and increases rapidly with the applied stimulus intensity. Our hypothesis is that these two distinct signals reflect the individual responses of rod and cone photoreceptors.

To facilitate a more rigorous quantitative investigation of this process, a composite impulse response, represented as Δ*l*(*t*), is fitted to the measured data, taking the form:


(1) $$\text{Δ}l\left(t\right)=a_{1}t\times \text{exp}\left(-b_{1}t-c_{1}t^{2}-d_{1}t^{3}\right)+a_{2}t\times \text{exp}\left(-b_{2}t-d_{2}t^{3}\right)$$

Here, *a*_1_,_2_ are parameters governing the respective initial slopes, while *b*_1_,_2_ *,c*_1_ and *d*_1_,_2_ shape the duration of the rising and falling phases of the curves. Notably, the quadratic term associated with the slower response has been omitted from this fitting function due to its perceived redundancy.

**Figure 3:**
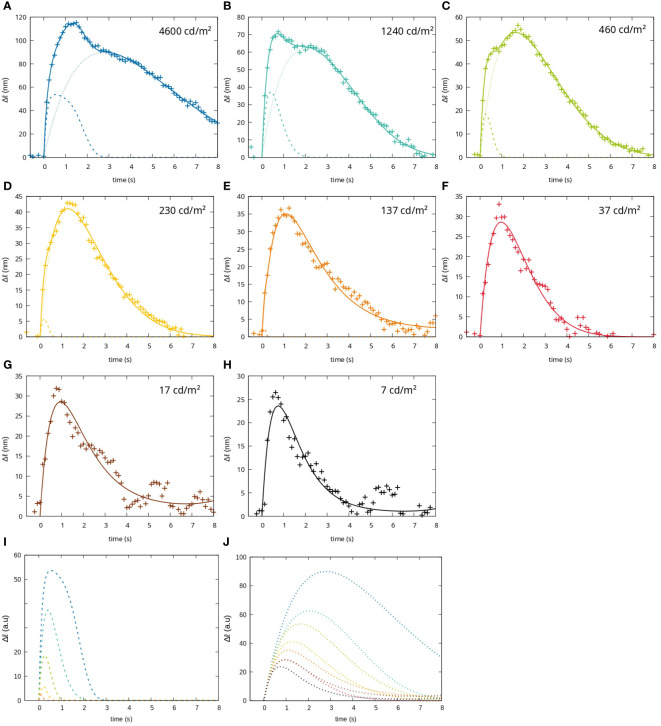
Fit of the functional signals to a short stimulus (100ms) with different intensities. The measured change in optical path length (crosses), the fit curve describing the signal as the sum of two impulse responses (solid lines), the impulse response attributed to rods (dotted line) and the impulse response attributed to cones (dashed lines) are shown for **(A)** 4600 $\text{cd}/{\text{m}}^{2}$ **(B)** 1240 $\text{cd}/{\text{m}}^{2}$ **(C)** 460 $\text{cd}/{\text{m}}^{2}$ **(D)** 230 $\text{cd}/{\text{m}}^{2}$ **(E)** 137 $\text{cd}/{\text{m}}^{2}$ **(F)** 37 $\text{cd}/{\text{m}}^{2}$ **(G)** 17 $\text{cd}/{\text{m}}^{2}$ and **(H)** 7 $\text{cd}/{\text{m}}^{2}$ , **(I)** cone fit-function for all luminances, **(J)** rod fit functions for all luminaces.

**Figure 4:**
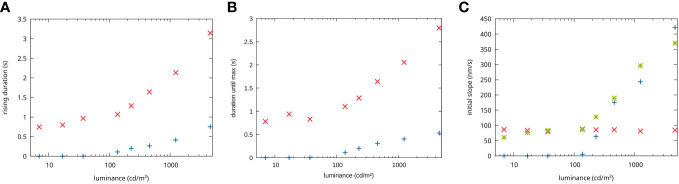
Dependencies on the stimulation intensities. **(A)** Duration until the maximum is reached in dependency of the stimulation luminance for the first impulse response attributed to rods (red) and the second impulse response attributed to cones (blue) signal. **(B)** Maximum amplitude reached for the rods (red) and cones (blue) impulse response. **(C)** Initial slope of the rods (red) and the cones (blue) fit responses, (green) actual measured slope.

This two-impulse-response model describes the measured changes in optical path length very well for the different stimulation conditions (see **Figure 3**). Furthermore, the mathematical description of the measurement in Equation (1) can be used to study the dynamics of the impulse responses separately. For this, we investigated the initial slope of the two impulse responses and the time taken to reach the maximum change in optical path length for the different stimulation conditions (see **Figure 4**).

It can be seen that both impulse responses increase their duration similarly with increasing stimulation intensity. However, the first impulse response (dotted lines) has a significantly longer rise time than the second (dashed lines). This discrepancy becomes more pronounced at higher stimulation intensities (see **Figure 4A**).

In terms of their initial slope, the two impulse responses behave very differently. While the first impulse response shows no change in slope with stimulation intensity, the second shows a strong dependence, especially at higher intensities. At lower light intensities, the signal is hardly present, so that for light intensities between 37 cd*/*m^2^ and 7 cd*/*m^2^ a fit of the first impulse response alone is sufficient to describe the measured data (see **Figure 4B**).

## 3 Discussion and conclusion

The two processes, that cause the two impulse responses, can be interpreted as the physiological responses of rods and cones to the same stimulus. The second impulse response (dashed lines) can be interpreted as the physiological response of cones and the first impulse response (dotted lines) as the physiological response of rods. It should be noted, however, that the measured data do not correspond to an addition of the two impulse responses, but to their weighted mean value. The actual height of the impulse response is therefore scaled according to the proportional composition of rods and cones and their contribution to the scattering intensity of the analyzed image field. Since the measurements shown here were taken at about 10° periphery of the fovea, where approximately the same number of rods and cones are present (18) and assuming that rods and cones contribute equally to the overall backscattered signal, an identical scaling factor of about 1/2 can be assumed for both impulse responses. However, the analysis of the duration and sensitivity of each impulse response remains completely independent of the scaling factor and can therefore be easily analyzed using this model.

In principle, rods and cones use the same amplification cascade, but the individual amplification steps are inactivated much more quickly in cones than in rods (19). As a result, the physiological response in rods is slower than in cones, which means that it takes longer to reach the maximum response strength (see **Figure 4A**).

Furthermore, a difference in the initial slope of the two processes is observed (**Figure 4B**). The initial slope of the first process, which we attribute to rods, stays more or less constant, whereas the initial slope of the second process increases with the stronger stimulation. The above-mentioned longer amplification cascade in rods leads to a higher sensitivity but also an earlier saturation. The intensity range used here is in the transition region from scotopic to photopic vision (**Figure 1**). Accordingly, at stimulation levels below 100 cd*/*m^2^, we only observe a nearly saturated response of the rods with no change in slope for varying intensities. In contrast, the cone cascade is not yet sufficient to detect a change in optical path length at low stimulation intensities. At higher intensities, however, the cone response becomes strong enough to be detected. The light levels used here are within the dynamic range of the cones, the initial slope of the impulse response increases with increasing intensity. Obviously, the initial slope is connected to the sensitivity of rods and cones to different stimulus intensities. In our earlier work, we accordingly observed a saturation of the initial slope of the whole ORG signal at higher stimulation levels (9).

In this study, we have demonstrated the separation of rod and cone signaling in ORG measurements with FF-SS-OCT by introducing a simple mathematical method that exploits the different temporal dynamics of the two cell types. Previous work on functional imaging was either unable to distinguish the functional contributions of rods and cones, or relied on technically complex and expensive setups to distinguish the functional changes of individual rod photoreceptor cells (8). FF-SS-OCT avoids the technical complexity otherwise required. In addition, due to its high sensitivity, FF-SS-OCT provides further information on the IPL functionality (12), which allows investigation of possible effects on the downstream cell levels. The time courses of functional changes in rods and cones determined in this work are in good qualitative agreement with the measured time courses of individual rods previously reported by Azimipour et al. (14). The response of the rods to the same stimulus lasts 4–5 times longer than that of the cones. Although the duration of individual impulse responses at comparable stimulus intensities differs between Azimipour et al. and the measurements shown here, this may be due to individual variability and a higher background stimulus and hence incomplete dark adaptation by the OCT system used here. The stimulation intensity at which the cone signal can be detected for the first time in the ORG is here at approx. 0.2% photopigment bleaching. This corresponds approximately to our illuminance of 230 cd*/*m^2^ and is also the point at which a cone signal can be detected for the first time. Similarly, Azimipour et al. (8) also show a strong dependence of the initial slope in cones on the mesoscopic stimulation intensity. A different behavior is observed for rods, as Azimipour et al. also observed a change in slope for different stimulation intensities, whereas no change is observed in the measurements shown here. However, these different observations do not necessarily contradict each other but could represent the response of rods to different stimulus intensities. For example, it has already been shown for the cone response that no change in slope can be observed at very high stimulation intensities (9). At the intensities we used, the rod photocurrent should be largely saturated, which is why we do not observe a change in slope. However, at lower intensities, corresponding to the dynamic range of the rods, a change in slope would be expected, as in the case of Azimipour. This transition is already visible at the lowest stimulation intensities used here (37 to 7 cd/m²), the rise time of the rods and the maxima recorded no longer change, while a slight change in the measured slope (**Figure 4C**, green dots) can be observed with the stimulation intensity. It can therefore still be assumed that the same process is observed. This indicates that the mathematical model developed here accurately describes the actual functional changes in the rods, so that physiological parameters such as sensitivity to different light intensities can be used to detect clinical changes in rod functionality.

A fundamental limitation of further investigations of rod signals in the scotopic range is the background stimulation by the light source of the OCT system. Although the light source used here with a center wavelength of 840nm is already at the edge of the visible spectrum, it causes a luminance of 2.2 cd*/*m^2^. At this luminance sufficient cones are still stimulated so that the light source can be perceived as red by the subject.

In addition, further measurements in other subjects are needed to confirm the results shown here. It would also be interesting to investigate in future studies how the preferential stimulation of S-/M- and L-cones affects the signal to draw possible conclusions about the distribution of the different cones in different subjects.

Many retinal conditions, such as retinitis pigmentosa and certain forms of macular degeneration, exhibit complex patterns of photoreceptor dysfunction, with rods and cones being affected in unique ways. E.g., there is evidence that early age-related macular degeneration (AMD) is linked to changes in rod and cone function. Especially the dark adaption (20, 21) and scotopic microperimetry was proposed for an early diagnosis (22, 23). Objective functional testing of rod and cone function by ORG could be a new option for early AMD diagnosis. The ability to separate and analyze the functional contributions of rods and cones using the method described may provide valuable insights into the progression and severity of these diseases. This, in turn, may lead to more accurate and sensitive diagnostic tools, ultimately benefiting individuals with retinal disorders and aiding in the development of targeted therapeutic interventions.

## 4 Measurements

**Figure 5:**
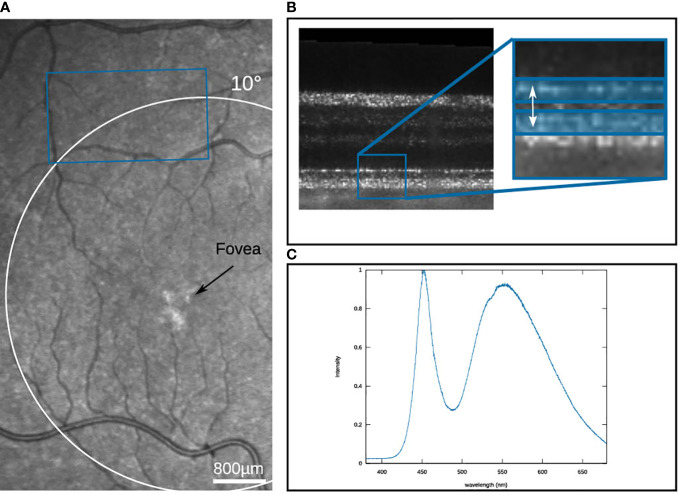
**(A)** SLO image of the volunteer’s eye with the measured area marked in blue. **(B)** B-scan of the measured region, with the two reference layers marked in blue **(C)** Spectrum of the stimulation LED.

For the measurement, the retina of a volunteer was imaged by a Michelson interferometer-based Full-Field Swept-Source OCT system (FF-SS-OCT) [detailed description can be found here (12)] using a tunable light source (Superlum Broadsweeper, BS-840-1) with 51nm tuning range centered at *λ*_0_ = 841nm. The light source illuminates an area of 2.6 mm × 1.5 mm on the retina, which is detected by a high-speed camera (Fastcam SA-Z, Photron) at a framerate of 60 kHz. The detected raw volume size corresponds to 640×364×512 px. For the volume size, the number of volumes per measurement is limited to 70 volumes, by the data storage capacity of the camera. To ensure longer measurement times, a volume is only triggered every 125 ms, resulting in a total measurement duration of 8.625 s. For the OCT imaging, the irradiance on the retina was 5.2 mW, which corresponds to a luminance of 2.2 cd*/*m^2^, which is in the lower mesoscopic range. The measurements were performed on a healthy volunteer with no known diseases or ametropia. Written informed consent was obtained from the subject. Compliance with the maximum permissible exposure of the retina and all relevant safety rules were confirmed by the ethics board of the Universität zu Lübeck. To improve the image quality, the volunteer’s pupil was dilated with eye drops to a diameter of approximately 8 mm. Before the measurement, the volunteer was dark adapted for 20 min. After each measurement, a break of at least five minutes was taken to obtain a full regeneration of the stimulated area. The image field was selected from a previously captured SLO image so that the stimulation took place in the 3 mm periphery, and at the same time, there were no larger vessels in the immediate vicinity that could influence the evaluation due to their pulsation (see **Figure 5A**). The selected position was controlled by a static fixation target during the OCT measurement. For the evaluation, the OCT data were numerically corrected for dispersion and axial motion (24). Furthermore, the movements occurring between successive volumes are corrected by a co-registration of the data. Further, the IS/OS was segmented and referenced to a parallel layer 4 pixels below. To improve the SNR of the phase evaluation, both layers were averaged over a depth of 3 pixels (as shown in **Figure 5B**), so that it can be assumed that the contributions of rods and cones are equally included in the evaluation. The phase evaluation is adapted from the extended-Knox-Thompson algorithm according to (25).

The conversion from the irradiance *E_e_* [W*/*m^2^] to a photometric unit illuminance *E_v_* [lm*/*m^2^] is done by the following equation.


(2) $$E_{v}=683\;\frac{\text{lm}}{\text{W}}\int \text{d}\lambda \;V\left(\lambda \right)E_{e}\left(\lambda \right)$$

Here $683\frac{\text{lm}}{\text{W}}$ is a conversion factor, for the definition of lumen, and *V* (*λ*) is the luminous efficiency function, which describes the relative spectral sensitivity of the eye (26). Using the spectrum of the light-emitting diode (LED) ${\text{Φ}}_{e}\left(\lambda \right)$ (shown in **Figure 5C**), the integral $\int \text{d}\lambda V\left(\lambda \right){\text{Φ}}_{e}\left(\lambda \right)$ from Equation (2) confers an efficiency factor of 0.61. However, since it is difficult in most cases to determine illuminance on the retina directly, the luminance $L_{v}\;\left[\text{cd}/{\text{m}}^{2}\right]\;$ is used in this work. The illuminace at the retina is connected with the luminance at the cornea via the pupil area *A_p_* = 50.26 mm^2^, predicated upon a maximal pupil diameter of 8 mm, and the distance from the pupil to the retina *D* = 17 mm according to:


(3) $$L_{v}={\text{Φ}}_{v}\frac{D^{2}}{A_{p}}$$

The converted values for the different stimuli are shown in **Table 1** as well as the number of photons per *µ*m^2^ and the percentage of photopigment bleach within one second of stimulation in rods. Again a stimulation efficiency factor of 0.61 was assumed, a base area for rods of *A*_rod_ = 3.14 *µ*m^2^, and an absolute photopigment number of *N*_rod_ = 10^8^ (27). However, it must be noted that in the measurements of photopic and scotopic vision, the pupil size in Equation (3) would have in general adapted to the prevailing light conditions. This means that for high irradiances the pupil diameter would have decreased to approximately 2.5 mm, in order to reduce the incidence of light on the retina. In the measurements performed here, however, a blocking of the pupil reflex ensures that the pupil diameter is constant at 8 mm. While this makes little difference for the lower illuminances, where the pupil diameter is still relatively large, the highest luminance (4600 cd*/*m^2^) would have to be multiplied by a factor of 10 to be comparable to the measurements of (15). This is indicated by the dashed rectangle in **Figure 1**. Additionally, the luminance caused by the broad sweeper of the OCT imaging was calculated. For this, an intensity of 0.83 mW*/*mm^2^ is used. Since for the central wavelength *λ*_0 _= 840 nm, *V*(*λ*) is not defined anymore, the closest available wavelength (830 nm) is used for the calculation with *V* (830 nm) = 6.6 × 10^−7^ (26).

## Data availability statement

The raw data supporting the conclusions of this article will be made available by the authors, without undue reservation.

## Ethics statement

The studies involving humans were approved by ethics board of the Universität zu Lübeck. The studies were conducted in accordance with the local legislation and institutional requirements. The participants provided their written informed consent to participate in this study. Written informed consent was obtained from the individual(s) for the publication of any potentially identifiable images or data included in this article.

## Author contributions

CP: Data curation, Investigation, Software, Visualization, Writing – original draft. LP: Data curation, Validation, Writing – review & editing. HS: Data curation, Validation, Writing – review & editing, Investigation. DH: Data curation, Funding acquisition, Software, Validation, Writing – review & editing, Investigation. YM: Supervision, Validation, Writing – review & editing. GH: Funding acquisition, Project administration, Supervision, Validation, Writing – review & editing.
